# Lung Interstitial Macrophages: Past, Present, and Future

**DOI:** 10.1155/2018/5160794

**Published:** 2018-04-30

**Authors:** Joey Schyns, Fabrice Bureau, Thomas Marichal

**Affiliations:** ^1^Laboratory of Cellular and Molecular Immunology, GIGA-Research, University of Liège, 4000 Liège, Belgium; ^2^Faculty of Veterinary Medicine, University of Liège, 4000 Liège, Belgium; ^3^Walloon Excellence in Life Sciences and Biotechnology (WELBIO), Wallonia, Belgium

## Abstract

For a long time, investigations about the lung myeloid compartment have been mainly limited to the macrophages located within the airways, that is, the well-known alveolar macrophages specialized in recycling of surfactant molecules and removal of debris. However, a growing number of reports have highlighted the complexity of the lung myeloid compartment, which also encompass different subsets of dendritic cells, tissue monocytes, and nonalveolar macrophages, called interstitial macrophages (IM). Recent evidence supports that, in mice, IM perform important immune functions, including the maintenance of lung homeostasis and prevention of immune-mediated allergic airway inflammation. In this article, we describe lung IM from a historical perspective and we review current knowledge on their characteristics, ontogeny, and functions, mostly in rodents. Finally, we emphasize some important future challenges for the field.

## 1. From Septal Cells to Interstitial Macrophages

Phagocytic “septal cells” were observed by Kaplan and colleagues already in 1950 [1] and likely represented “nonalveolar” macrophages located in the alveolar wall. Nevertheless, the alveolar macrophages (AM) remained the main macrophage population investigated in the lung until the early 1970s. By that time, it was proposed by van Furth and Cohn that, like any other tissue-resident macrophages, AM originated from bone marrow promonocyte precursors, which then circulated in the blood as monocytes and could differentiate into macrophages within the alveoli [2]. As a corollary, an intermediate state of AM maturation, located in the pulmonary interstitium, presumably existed between the blood compartment and the airways. In 1972, “mononuclear interstitial cells” were first proposed as precursors of the AM lineage in cultured lung explants [3]. Since then, lung tissue macrophages were long merely considered as a transition state between circulating monocytes and AM [4–6].

The development of methods to harvest pulmonary macrophages using mechanical and enzymatic treatments allowed the comparison between AM (isolated by bronchoalveolar lavage (BAL)) and lung tissue macrophages (TM) in rodents, even though the latter were contaminated by residual AM [7, 8]. While both AM and TM displayed classical macrophage features such as a phagocytic potential and expression of Fc receptors, these features were reduced in TM as compared to AM [9–13]. Moreover, additional differences were underscored within TM. In mice, TM exhibited a higher percentage of cells positive for the complement receptor C3 [8, 9], a higher production of arachidonic acid metabolites following phagocytosis [14], and an increased spreading capacity when exposed to plasma [9] as compared to AM. In rats, TM were shown to have a higher peroxidase activity [15], a greater major histocompatibility complex class II (MHC-II) expression [16], and a greater number of filopodia [17]. Upon ex vivo stimulation with lipopolysaccharide (LPS), AM displayed greater cytotoxic and antimicrobial activities than TM, while TM secreted more interleukin- (IL-) 1 and IL-6, in mice [13] and rats [16]. Unlike AM, mouse TM were also very potent in promoting mitogen-stimulated spleen lymphocyte proliferation in mice [13]. Despite these morphological, phenotypical, and functional differences, many authors still interpreted them as being part of the transition process between blood-circulating monocytes and AM [3–6, 13], but others raised the possibility that lung TM (also called interstitial macrophages (IM)) represented a distinct and fully competent macrophage population [11, 16, 18], a concept that is now well accepted in the field [19–21].

## 2. Morphological and Phenotypical Features

Most of the abovementioned studies have been performed ex vivo and have defined TM as the cells collected from enzymatically digested lungs and adherent to the culture plate in vitro. Obviously, such a technique did not allow a specific isolation of IM, and the resulting cells were likely contaminated with variable amounts of other mononuclear cell types, such as residual AM (despite extensive BAL [11, 22–24]), conventional dendritic cells (cDCs), or monocytes [25, 26]. In addition, accepting that macrophages, once extracted from their native microenvironment and cultured ex vivo, undergo rapid morphological and phenotypical changes [27], the conclusions drawn from ex vivo-cultured IM have to be interpreted with caution.

Morphologically, Sebring and Lehnert were the first, to our knowledge, to combine a Fc receptor-based affinity technique with a cytometric approach to sort IM from rat lungs and identified them as being smaller than AM, with a smoother surface and a more irregular and heterochromatin-containing nucleus [28]. More recently, freshly isolated mouse IM were shown to exhibit an irregularly shaped nucleus and numerous vacuoles in their cytoplasm, while mouse AM were larger cells [26] with more prominent pseudopodia [29].

**Figure 1:**
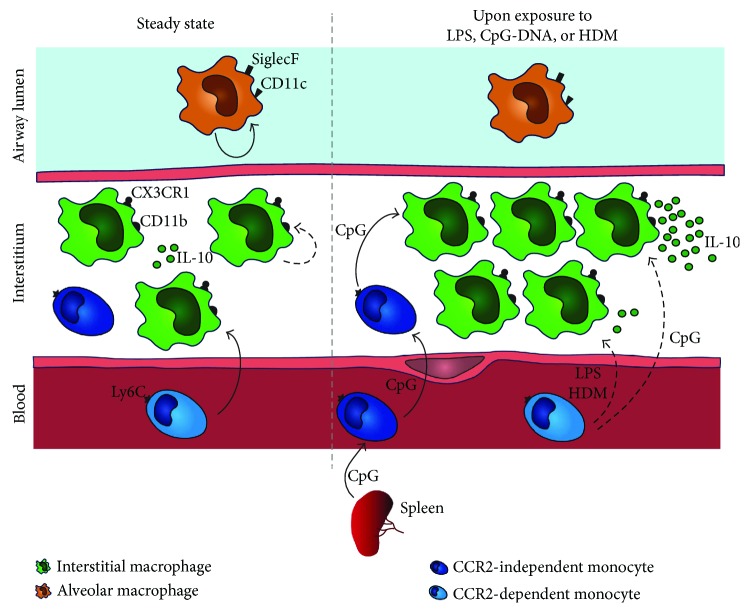
Mouse lung interstitial macrophage phenotype and origin at the steady state and upon exposure to LPS, CpG-DNA, or HDM. For clarity, the ratio between the numbers of depicted AM, IM, and monocytes does not reflect the reality. By definition, IM are located in the lung interstitium, while AM reside in the airway lumen. IM can produce IL-10 at baseline, a phenomenon that is potentiated by an exposure to LPS, CpG-DNA [26], or HDM [50]. Phenotypically, IM are non-autofluorescent SiglecF^−^CD11b^+^CX3CR1^+^Ly6C^−^ cells, while AM are autofluorescent SiglecF^+^CD11c^+^CD11b^−^CX3CR1^−^Ly6C^−^ cells. Steady-state IM, as well as LPS- or HDM-induced IM, are thought to be maintained or expanded by the recruitment of CCR2-dependent Ly6C^+^ classical blood monocytes, at least in part. Local proliferation may also account for the maintenance of steady-state IM. Following exposure to CpG-DNA, CCR2-independent lung-resident and splenic Ly6C^+^ monocytes contribute to a large extent to the expansion of the IM pool endowed with enhanced immunoregulatory properties.

The availability of technologies allowing analysis of freshly isolated single cells, such as multicolor flow or mass cytometry, substantially improved the phenotypic characterization of lung immune cells [20, 25, 26, 29–31]. The work of several investigators in the field has allowed, based on the levels of expression of several surface markers, a discrimination between each of the lung myeloid mononuclear cell populations in the steady-state lung, including IM (Figure 1). These markers are compiled in Table 1. Both IM and AM express the macrophage-specific markers CD64 and Mertk, as opposed to cDCs and monocytes. While AM are autofluorescent SiglecF^+^CD11c^+^CD11b^−^CCR2^−^CX3CR1^−^ cells, IM are non-autofluorescent SiglecF^−^CD11c^+/−^CD11b^+^CCR2^+/−^CX3CR1^+^ cells [26, 31] (Figure 1). Notably, a recent report has shown that a fraction of mouse IM, defined as Mertk^+^CD64^+^CD11b^+^SiglecF^−^ cells, expressed CD11c and MHC-II [31], like cDCs, so that both cell types may potentially contaminate each other. Nevertheless, cDCs differ from IM by their low or absent expression of macrophage markers (e.g., CD64, Mertk, and F4/80). The situation may be more confusing when inflammation is present and monocyte-derived cells are infiltrating the lung, in which case IM may be included in inflammatory subtypes of monocytes or DCs. Nevertheless, IM may be discriminated from such cells by their low expression of the inflammatory/classical monocyte marker Ly6C.

## 3. Tissue Localization

At steady state, lung IM are primarily considered as “nonalveolar” macrophages and are therefore virtually absent in the airways, while AM represent the macrophages present in the airway lumen. To date, however, it must be noted that information about the exact localization of IM within the lung tissue remains scarce and is based on standard immunohistochemical procedures using nonspecific pan-macrophage markers [31, 32]. Earlier studies in mice using immunostainings against F4/80 and CD11c markers identified F4/80^+^CD11c^−^ cells, defined as IM, within the lung parenchyma, whereas AM, defined as F4/80^+^CD11c^+^ cells, were mostly located in the lumen [32]. Experiments using intravenous injection of clodronate-containing liposomes, which efficiently depleted blood monocytes, had no impact on IM numbers [26], supporting that steady-state IM were not associated with blood vessels but truly located in the lung tissue. More recently, Gibbings and colleagues have performed a staining for Mertk on mouse lung sections from CX3CR1-GFP reporter mice at steady state, allowing the visualization of Mertk^+^CX3CR1^+^ IM in the bronchial interstitium, in the vicinity of lymphatic vessels, but not in the lung parenchyma [31]. In the same report, no Mertk^+^CX3CR1^+^ cells were observed on the pleural surface nor in the blood vessels [31]. Given the complexity and dynamic regulation of the lung tissue macrophage compartment, the generation of novel transgenic tools allowing the specific tracking and visualization of IM in vivo will help solve the question of their localization and their spatiotemporal relationships with the local microenvironment, such as bronchial and alveolar epithelial cells, stromal cells, endothelial cells, or lymphoid tissues [33], at steady state and during inflammation. The fact that distinct subpopulations of IM exist, as reported recently [31], is consistent with the idea that they may reside in more than one anatomical site.

## 4. Origin, Maintenance, and Expansion

Most tissue-resident macrophages are thought to derive from embryonic precursors arising from different sources: the yolk sac (i.e., erythromyeloid progenitor- (EMP-) derived premacrophages), the fetal liver (i.e., EMP-derived monocytes or hematopoietic stem cell- (HSC-) derived monocytes), or the bone marrow (i.e., HSC-derived monocytes), as reviewed and discussed extensively elsewhere [34–36]. At steady state, the well-characterized AM have been shown, in mice, to originate from fetal monocytes that seed the airway lumen around birth [20]. On the contrary, IM ontogeny seems more complicated and less documented. In 2016, Tan and Krasnow investigated the development of lung macrophages by marker expression patterns and genetic lineage tracing [21]. The authors used *Runx1^CreER^* transgenic mice expressing the tamoxifen-inducible Cre recombinase under the control of the *Runx1* promoter (*Runx1* being expressed in primitive hematopoietic cells located exclusively in the yolk sac between E7 and E8 [37, 38]) and found that a subset of yolk-sac-derived premacrophages seeded the lung starting at E10.5 and persisted as “primitive” IM at specific submesothelial and perivascular locations in adults [21]. In addition, they identified an additional wave that developed rapidly after birth to give rise to “definitive” IM located diffusely in the lung parenchyma and thought to originate from the bone marrow [21]. These results are consistent with the idea that IM have a mixed origin, both an embryonic yolk-sac-derived origin and a postnatal bone marrow-derived origin.

During homeostasis, most embryonically derived tissue-resident macrophages, like AM, can self-maintain throughout life with minimal contribution from circulating monocytes [20, 35, 39–41]. In the case of IM, parabiosis studies have suggested that they are, at least in part, replenished from blood monocytes for their maintenance in adults [21, 26] (Figure 1), like macrophages from the intestinal lamina propria [42], skin [43], and heart [44]. In the report of Tan and Krasnow, parabiotic wild-type (WT) mice were sutured together and exchanged their circulation with “donor” ubiquitous EGFP mice for 4 months. The lungs of WT mice were then examined for enrichment in EGFP^+^ cells, and 17% of IM were EGFP^+^, demonstrating that circulating precursors can maintain the IM pool in adults, as opposed to AM [21]. Further supporting this, our group has analyzed the lungs of parabiotic *Ccr2^−/−^* mice (in which the egress of monocytes from the bone marrow is compromised [45]) that were sutured together with a WT “donor” for 6 months and showed that 35% of IM derived from WT cells. The relatively low percentage of IM replacement by circulating “donor” cells in parabiotic studies is consistent with the mixed origin proposed by Tan and Krasnow [21] and with the idea that only one subpopulation of IM is maintained by circulating monocytes after birth, whereas another subpopulation is long-lived and may be able to self-renew in the tissue (Figure 1). This idea is further supported by the study of Gibbings and colleagues identifying, in the mouse steady-state lung, at least three IM subsets, with one subset displaying a higher turnover rate and replenishment by circulating precursors than the two others [31].

Which population is preponderant in young, adult, and aged animals, which consequences does it have on their biological functions, and how is it influenced by the numerous immune challenges to which the lung is exposed throughout life remain interesting open questions for future research. Emphasizing the complexity of IM ontogeny in response to environmental stimuli, our group reported that local exposure to unmethylated CpG-rich DNA (CpG-DNA) promoted a robust TLR-9-dependent expansion of IM unexpectedly originating from monocytes residing in the lung or recruited from the spleen, independently of CCR2 [26] (Figure 1).

## 5. Heterogeneity and Plasticity

The existence of subpopulations of IM in rats was first proposed in 1986 by Chandler and colleagues [15] and further investigated by the same group [46, 47], based on density gradient fractionation. The fractions of lower density displayed greater functional capacities (e.g., Fc-mediated binding and phagocytic activity, production of prostaglandin and thromboxane, and migration upon exposure to chemotactic stimuli) as compared to the fractions of higher density [15, 46, 47]. However, it is unclear whether these differences may be attributed to a true heterogeneity within IM or to a contamination of the higher density fractions with granulocytes, as reported [15]. Nevertheless, several recent reports have provided experimental evidence that IM represented a heterogeneous population in the steady-state lung. First, the use of IL-10 reporter ITIB mice [48] supported that two subpopulations of IM exist in terms of IL-10 expression [26]. Second, IM have been shown to segregate in a phagocytic and a nonphagocytic compartment in vivo [49]. Third, as stated above, Gibbings and colleagues have recently described three distinct IM subpopulations, based on their relative surface expression of CD11c and MHC-II, namely, CD11c^low^MHC-II^low^ (IM1), CD11c^low^MHC-II^high^ (IM2), and CD11c^+^MHC-II^high^ (IM3) [31]. Phenotypically, IM1 and IM2 expressed higher levels of CD206, Lyve-1, and CD169 as compared to IM3, which expressed higher levels of CCR2 and CD11c. Functionally, IM1 and IM2 appeared to be more efficient than IM3 but less efficient than AM in the phagocytosis of latex microbeads or microbial bioparticles in vivo, whereas the three populations had similar phagocytic abilities when the experiment was performed ex vivo to provide a similar access to the beads for each subset [31].

These results highlight the potential diversity of mouse IM at steady state. It is very likely that the picture becomes even more complex when the lungs are exposed to endogenous or exogenous stress signals, such as following tissue damage or during inflammation or infection. Under these circumstances, IM may adapt their phenotype and function to respond to the needs of the lung tissue, and additional inflammatory monocytes may also be recruited into the lung and acquire features of IM. Supporting this, Kawano and colleagues have shown that the numbers of IL-10-producing IM were increased following local challenge with house dust mite extracts (HDM) in mice [50]. In addition, we have shown that local exposure of mice to LPS or CpG (i.e., ligands of the TLR-4 and TLR-9, resp.) induced increases in IM numbers as well as substantial phenotypical changes, while no change in IM numbers was detected in response to lung infection with influenza A virus or *Staphylococcus pneumoniae*, or following intranasal exposure to ligands of TLR-1/2, TLR-3, and TLR-2/6 [26]. Besides microbial products, IM may also be impacted by tissue damage and hypoxia [29, 51, 52]. Indeed, increases in IM numbers have also been observed in mouse models of acute lung injury (ALI) based on local instillations of bleomycin [29] or high doses of LPS [51]. Such IM expressed higher levels of classically activated “M1” macrophage markers (CD40, CD80, and CD86) as compared to basal IM. During the later stages of tissue repair, however, IM numbers and phenotype returned to baseline levels [51]. In response to low oxygen levels in mice, numbers of IM transiently increased and their transcriptome seemed to shift toward an anti-inflammatory gene profile at a later stage [52], consistent with a previous observation that the hypoxia-responsive transcription factor Hif1*α* promoted IM immunoregulatory activity in allergenic contexts [53].

## 6. Biological Functions *In Vivo*

Many putative functions of IM in vivo could be speculated based on their phenotypical and functional properties. Like AM, IM are phagocytic cells [14–16, 31, 32, 49, 54] and could thus be considered as a second line of defense against invading microorganisms. In addition, based on their expression of MHC-II [26, 31, 54], one can postulate that mouse IM could exhibit some antigen-presenting cell activity, as suggested by earlier reports [55]. So far, however, most of the functional studies on IM in mice focused on their potential immunoregulatory properties. Indeed, mouse and human IM have been shown to express the immunosuppressive cytokine IL-10 at steady state [24–26, 32, 56] (Figure 1). Such IL-10 expression increases in response to environmental stimuli such as LPS, CpG-DNA, or HDM [26, 50, 56] (Figure 1). Knowing that the lung mucosa is constantly exposed to a wide range of immunostimulatory molecules and allergens, we postulated that IM may contribute to lung homeostasis through the alteration of lung cDC functions, which are endowed with the ability to trigger an allergen-specific T helper type 2 (Th2) cell response orchestrating the development of allergic airway inflammation in mice exposed to LPS and allergens [57–60].

Using a coculture system between freshly isolated IM and LPS- and ovalbumin- (OVA-) pulsed bone marrow-derived DCs (BMDCs) in vitro, IM were found to impair the ability of BMDCs to migrate to the draining lymph node and to induce features of Th2-mediated airway allergy once reinjected in the trachea of recipient mice through TLR-4-, HIF1*α*-, and IL-10-dependent mechanisms [32]. Notably, while isolated and cocultured IM may have encompassed other cell types such as F4/80-expressing monocytes or resident eosinophils [26, 61], the “true” IM were the only cells able to secrete IL-10, and the ability of FACS-sorted pure IM to inhibit DC function has been confirmed later [26, 61]. In vivo, systemic treatment of WT mice with depleting antibodies directed against F4/80 induced a depletion of IM, but not AM, and triggered increased activation of lung cDCs and the development of overt Th2 and allergic airway inflammation when mice were exposed to low doses of an allergen/LPS mixture [32], further supporting a tolerogenic role for IM in maintaining lung homeostasis.

Mouse IM may be implicated in the control not only of allergic asthma in mice but also of other asthma phenotypes. Indeed, Kawano and colleagues have provided evidence that IM contributed to the prevention of Th17-mediated neutrophilic airway inflammation by IL-10-dependent mechanisms [50]. They used a neutrophilic asthma model based on HDM instillations in *Il10^−/−^* mice, which dramatically increased the number of neutrophils in the BAL fluid and promoted lung neutrophilic infiltration and expression of Th17-related cytokines as compared to HDM-exposed WT mice. In this model, they showed that the transfer of WT IM in *Il10^−/−^* mice before the HDM challenge could inhibit the neutrophilic inflammation and mucus production, which was associated with a decrease of Th17-related cytokines and IL-13 [50].

The fact that IM respond to LPS and CpG-DNA, two bacterial products omnipresent in the environment [62, 63], suggests a link with the “hygiene hypothesis,” which postulates that decreased exposure to environmental and commensal microbes or their products (PAMPs), partly because of changes associated with urban lifestyles, is responsible for the dramatic increase in the prevalence of allergies and asthma over the past decades [64, 65]. In line with this assumption, several epidemiological studies have demonstrated that growing up on a farm, where exposure to environmental and commensal PAMPs is high, reduces the risk of allergic sensitization [62, 65]. Exposure of humans or mice to CpG-DNA from bacteria reproduces the*s*e protective effects [66–71], suggesting a contribution of CpG-DNA to microbe-induced asthma resistance. In mouse models, local CpG-DNA exposure had the unique ability to amplify the IM pool from monocytes residing in the lung or recruited from the spleen, which acquired a hypersuppressive profile [26]. Importantly, such CpG-DNA-induced IM were suggested to mediate the protective effects of CpG-DNA on allergic airway sensitization and inflammation, since adoptive transfer of IM isolated from CpG-DNA-treated WT mice, unlike the *Il10^−/−^* counterparts, recapitulated the effects of CpG when administered before allergen sensitization or challenge [26]. While speculative at this point, these findings provide a possible mechanistic explanation for the reduced risk of asthma in a microbe-rich environment and for the immunotherapeutic effects of synthetic CpG-DNA in experimental models and human clinical trials.

## 7. IM in Human and Nonhuman Primates

IM were already observed more than three decades ago in lungs from healthy subjects [72] or diseased patients [73, 74]. By that time, the functional studies comparing human lung macrophages obtained from the BAL and from the whole lung revealed very few differences between BAL-derived AM and tissue IM, possibly because tissue macrophages were heavily contaminated by residual AM [74].

Like in rodents, IM isolated from minced and digested human lungs are smaller and more heterogeneous in shape [54, 56], displayed a lower phagocytic activity [54], and expressed more surface MHC-II (HLA-DR) [56, 75] as compared to AM. Lung IM, which were obtained from the uninvolved lung tissue of patients undergoing a surgical resection for lung carcinoma and put in contact with stimulated T cell membranes, produced higher levels of matrix metalloproteinases (MMPs) and of an inhibitor of MMP (TIMP-1), whereas AM did not significantly react under the same conditions [75], suggesting a possible contribution of human IM in the regulation of lung tissue remodeling.

Hoppstädter and colleagues showed that the secreted levels of IL-6, IL-10, and IL-1 receptor antagonist (IL-1Ra) were higher in IM than in AM, both at baseline and after stimulation with LPS [56]. *Il10* gene expression was also higher in IM at baseline and after stimulation with LPS or DNA from certain bacteria [56], reminiscent of what is observed in mice. On the contrary, AM secreted higher levels of proinflammatory cytokines, such as IL-1*β*, IFN-*γ*, IL-12p40, or IL-12p70, after LPS stimulation [56]. Altogether, these results supported a more pronounced anti-inflammatory phenotype of IM as compared to AM and are consistent with a potential role for IL-10-producing IM in the maintenance of lung homeostasis in humans. Interestingly, a recent study performed on bronchial biopsies of asthmatic patients and healthy subjects showed that asthmatic airways were characterized by less IL-10^+^ IM as compared with healthy airways, suggesting that IM may be functionally impaired in asthma [76].

Given the limited access to healthy human samples, Cai and colleagues performed some studies on rhesus macaques as a model to unravel the human lung macrophage identity and diversity [5]. IM were defined as HLA-DR^high^CD206^−/int^CD11b^high^ cells and were located in the peribronchovascular and subpleural regions, whereas AMs were defined as CD206^+^CD11b^int^ larger cells that were located almost exclusively in the alveoli. Notably, the IM population had probably been confounded with tissue monocytes in this study, since IM and CD14^+^ blood monocytes resembled each other when analyzing the expression of 27 different markers, with the exception of CCR2 being highly expressed by blood monocytes and poorly by IM. This could also account for the fact that “IM” were found positive for BrdU as soon as 48 hours after its intravenous injection and were thought to contribute to the repopulation of AM after BAL-induced depletion [5].

Recent reports are aimed at identifying markers to discriminate IM from other lung monocyte and macrophage populations in human pulmonary tissue. In humans, AM and monocytes can be defined as highly autofluorescent SSC^hi^CD169^hi^CD206^hi^ and SSC^lo^CD169^−^CD206^−^CD14^+^CD16^lo/hi^ cells, respectively [77–79]. In addition, a population of HLA-DR^+^CD169^lo^CD206^int^ cells was identified in the human lung, whose size was intermediate between AM and monocytes [77–79] and which may correspond to human IM [77, 79]. Functional studies of these cells could help in determining their homology with murine IM. Human lung macrophages of COPD patients were also characterized recently [80]. In this report, “IM” were shown to be divided into two subpopulations: a scarce population of large macrophages, which expressed more CD206, and a population of small macrophages, potentially monocyte-derived, expressing more HLA-DR, CD14, CD38, CD36, and proinflammatory genes as compared to the other lung macrophages [80]. While this study has mainly focused on the analysis of pathological tissues, it emphasizes the complexity of the IM pool in diseased patients. It also suggests that IM can exert either anti- or proinflammatory properties, depending on the physiological or pathological conditions to which they are exposed.

## 8. Future Challenges

While substantial progress has been made regarding the ontogeny, phenotype, and functions of IM in mice, it is only the beginning of the story. First, to date, IM or IM subsets have been only characterized as bulk populations, defined according to a limited number of markers, thus revealing average signatures and ignoring the true and unbiased heterogeneity and structure of the populations of interest. There is therefore a need for an unbiased characterization of the IM population, both in mice and humans. Recent development and availability of high-dimensional single-cell technologies [81, 82] should help study highly diverse and heterogeneous immune cells such as IM.

Second, the biological responses modulated by IM or IM subpopulations in vivo remain rudimentarily investigated but are likely highly diverse and complex. This is partly due to the current lack of selective tools to track, modulate, or deplete IM (or IM subsets) in animal models, which will be instrumental in deciphering the biological functions of IM in health and diseases. On the one hand, IM may contribute to important physiological processes during lung development, metabolism, or aging. On the other hand, IM may also modulate several aspects of the pathological responses observed in lung chronic inflammatory disorders such as chronic obstructive pulmonary diseases (COPD).

Third, translational studies aimed at defining lung IM identity, heterogeneity, and functions in humans will be essential to find novel therapeutic targets for the prevention or treatment of lung diseases in which IM (dys)functions are, or will be, implicated.

## Figures and Tables

**Table 1:** Expression of the indicated surface markers between alveolar macrophages (AM), interstitial macrophages (IM), CD11b^+^ and CD103^+^ conventional dendritic cells (cDCs), and Ly6C^+^ and Ly6C^−^ monocytes (Mo) in the mouse lung at steady state.

Marker	AM	IM	CD11b^+^ cDCs (cDC2)	CD103^+^ cDCs (cDC1)	Ly6C^+^ Mo	Ly6C^−^ Mo
CCR2	− [26, 31]	+/− [26, 31]	− [31]	− [31]	+ [26, 31]	+/− [26]
CD103	− [29]	− [29]	− [29]	+ [29]	− [29]	− [29]
CD115	+/− [26, 31]	+/− [26, 31]	+ [31]	− [31]	+ [26, 31]	+ [26]
CD11b	− [10, 26, 29, 31, 50]	+ [10, 26, 29, 31, 50]	+ [29, 50]	− [29, 31]	+ [26, 29, 31]	+ [26, 29]
CD11c	+ [10, 26, 29, 31, 32, 50]	− [10, 32]; +/− [26, 31, 50]; + [29]	+ [10, 29, 31, 32]	+ [10, 29, 31, 32]	− [26, 29]; +/− [31]	+/− [26, 29]
CD14	+/− [29]	+ [29]	+/− [29]	− [29]	− [29]	− [29]
CD169	− [13]; + [26, 31]	+/− [13, 31]; + [26, 31]	− [31]	− [31]	− [26, 31]	− [26]
CD206	+ [29]	+/− [29]	+/− [29]	− [29, 31]	− [29, 31]	− [29]
CD24	− [29]	− [29]	+ [29]	+ [29]	− [29]	− [29]
CD36	+ [29, 31]	+ [29, 31]	− [31]; +/− [29]	+ [29, 31]	+/− [29]; + [31]	− [29]
CD64	+ [26, 29, 31]	+ [26, 29, 31]	− [29, 31]	− [29, 31]	− [26]; +/− [29, 31]	− [26]; +/− [29]
CD86	− [50]; +/− [26]	+ [26, 50]	− [50]		− [26]	− [26]
CX3CR1	− [26, 31]	+ [26, 31]	+/− [31]	− [31]	+/− [31]; + [26]	+ [26]
F4/80	+ [10, 26, 29, 31, 32, 50]	+ [10, 26, 29, 31, 32, 50]	− [10, 32, 50]; +/− [29]	− [10, 29, 31, 32]	+/− [31]; + [26, 29]	+ [26, 29]
Ly6C	− [26, 29]	− [26, 29]	+/− [29]	− [29]	+ [26, 29]	− [26, 29]
Lyve-1	− [31]	+/− [31]	− [31]	− [31]	− [31]	
Mertk	+ [26, 31]	+ [26, 31]	− [31]	− [31]	− [26, 31]	− [26]
MHC-II	+/− [13, 26, 29, 32]	+/− [31]; + [13, 26, 29, 32, 50]	+ [29, 31, 32, 50]	+ [26, 29, 31]	− [26, 29]; +/− [31]	− [26, 29]
SiglecF	+ [26, 29, 31]	− [26, 29, 31]	− [29, 31]	− [29, 31]	− [26, 29]	− [26, 29]
Zbtb46	− [31]	− [31]	+ [31]	+ [31]	− [31]	

−: absence of expression or low expression; +/−: intermediate or variable expression; +: high expression.
